# Cloning, expression and molecular characterization of a *Cystoisospora suis* specific uncharacterized merozoite protein

**DOI:** 10.1186/s13071-017-2003-1

**Published:** 2017-02-07

**Authors:** Aruna Shrestha, Nicola Palmieri, Ahmed Abd-Elfattah, Bärbel Ruttkowski, Marc Pagès, Anja Joachim

**Affiliations:** 1 Institute of Parasitology, Department of Pathobiology, University of Veterinary Medicine Vienna, Veterinaerplatz 1, Vienna, A-1210 Austria; 2 HIPRA, Amer, 17170 Spain

**Keywords:** Cystoisosporosis, Recombinant antigen, Invasion inhibition, Protozoa, Swine, Apicomplexa

## Abstract

**Background:**

The genome of the apicomplexan parasite *Cystoisospora suis* (syn. *Isospora suis*) has recently been sequenced and annotated, opening the possibility for the identification of novel therapeutic targets against cystoisosporosis. It was previously proposed that a 42 kDa uncharacterized merozoite protein, encoded by gene CSUI_005805, might be a relevant vaccine candidate due to its high immunogenic score, high expression level and species-specificity as determined *in silico*.

**Methods:**

The 1170 bp coding sequence of the CSUI_005805 gene was PCR amplified and cloned into the bacterial expression vector pQE-31. The specificity of the expressed recombinant protein was evaluated in an immunoblot, and relative levels of expression in different developmental stages and subcellular localization were determined by quantitative real-time PCR and indirect immunofluorescence assay, respectively.

**Results:**

The CSUI_005805 gene encoded for a 389 amino acid protein containing a histidine-rich region. Quantitative RT-PCR showed that CSUI_005805 was differentially expressed during the early development of *C. suis* in vitro, with higher transcript levels in merozoites compared to sporozoites. The recombinant protein was specifically recognized by sera from chicken immunized with recombinant CSUI_005805 protein and sera from piglets experimentally infected with *C. suis*, all of which suggested that despite prokaryotic expression, the recombinant CSUI_005805 protein maintained antigenic determinants and could elicit an immune response in the host. Immunofluorescence labelling and confocal microscopy revealed localization primarily at the surface of the parasite.

**Conclusions:**

The results suggest that CSUI_005805 is highly expressed in merozoites and might thus be critical for their survival and establishment inside host cells. Owing to its specificity, localization and expression pattern, CSUI_005805 could be exploited as an attractive candidate for alternative control strategies against *C. suis* such as vaccines.

**Electronic supplementary material:**

The online version of this article (doi:10.1186/s13071-017-2003-1) contains supplementary material, which is available to authorized users.

## Background

*Cystoisospora suis* (syn. *Isospora suis*), an enteric protozoan parasite of swine, is a member of the phylum Apicomplexa and the causative agent of neonatal porcine coccidiosis (cystoisosporosis). It is distributed worldwide with high prevalence rates in intensive pig breeding facilities regardless of the farm management system [1, 2]. Suckling piglets in the first 3 weeks of life are most prone to clinical disease, whereas the infection is usually asymptomatic in older piglets with little or no oocyst excretion [3, 4]. *Cystoisospora suis* has a direct life-cycle with faecal-oral transmission that facilitates its rapid spread among and between litter-mates [5]. Upon ingestion, sporulated oocysts undergo excystation and sporozoites then invade enterocytes to develop into merozoites [6] followed by gamonts [7, 8]. Infected piglets show watery to pasty non-hemorrhagic diarrhea [1], weight loss and uneven weaning weight [9–11] leading to significant economic losses for pig breeders [12, 13].

In the European Union, control of cystoisosporosis can currently be accomplished by metaphylactic medication with toltrazuril which is highly effective [11, 12, 14, 15]. However, emerging resistance is of concern as several incidences of drug resistance against anticoccidials including toltrazuril have already been reported in *Eimeria* of poultry [16, 17], which, along with legislative restrictions on the use of anticoccidials in many countries and increasing consumers’ interest in drug residue-free animal products, has led to an urge for development of alternative intervention strategies.

Live, virulent vaccines in large amounts, are impractical for the use in swine as even low infection doses can lead to disease in very young piglets [18], and attenuated lines have not yet been introduced for *C. suis*. An alternative approach would be the introduction of subunit or recombinant vaccines, which demands a systematic search for antigenic proteins to find appropriate vaccine candidates for testing.

The identification of protective antigens is vital for the development of any modern vaccine. In the closely related genus *Eimeria*, several attempts have been made to identify and characterize antigen-coding transcripts from relevant developmental stages [19–26]. The genome of *C. suis* has recently been sequenced and contains more than 11,000 protein-coding genes, most of which are expressed in merozoites. However, the functional annotation of coding sequences is still a major challenge. Indeed, more than 40% of the *C. suis* genes are currently categorized as of unknown function or annotated as “uncharacterized hypothetical proteins” [27]. The genes with unknown function that are considered unique to *C. suis* may be the most relevant ones to investigate as specific targets for recombinant vaccine development.

In a previous study, 399 (34%) of the 1168 potential vaccine candidates identified by screening of the predicted *C. suis* proteome also had no annotated function [27]. Homology-based searches indicated that a highly expressed protein of merozoites, encoded by the gene CSUI_005805 and with unknown function, also lacks orthologs in other organisms, making it an attractive target candidate for further research. In the current study, the complete coding region of the CSUI_005805 gene, encoding a novel *C. suis* specific protein, was cloned, expressed in *E. coli* and characterized in vitro. To date, this is the first attempt to identify and characterize a species-specific antigenic protein of *C. suis*. Based on immunolocalization and expression pattern of transcripts, it is suggested that this protein may be important for the growth and proliferation of merozoites inside host cells.

## Methods

### In vitro culture and parasite harvest

Merozoites of *C. suis* were maintained in intestinal porcine epithelial cells (IPEC-J2) as described elsewhere [28]. Free merozoites were harvested by collecting supernatant of the culture medium 5–6 days post-infection (p.i.), washed with phosphate buffer saline (PBS) and purified using a Percoll^®^ density gradient. Further, merozoites were filtered through Partec CellTrics^®^ disposable filters (50 μm), washed twice with PBS and pelleted by centrifugation at 1000× g for 10 min. Purified merozoites were snap frozen in liquid nitrogen and stored at -80 °C until further use.

### Crude merozoite lysate preparation

Crude lysate of purified merozoites in PBS was prepared by rapid freeze-thawing using liquid nitrogen followed by disruption in a TissueLyser II (Qiagen, Hilden, Germany). The preparation was then centrifuged at 20,000× g for 10 min at 4 °C to separate soluble and insoluble fractions. The insoluble fraction was dissolved separately in buffer with urea (7 M urea, 2 M thiuourea, 4% 3-((3-cholamidopropyl) dimethylammonio)-1-propanesulfonate, 1% (w/v) dithiothreitol, 20 mM Tris). Protein concentration was determined by a Bradford assay [29] using serial dilutions of bovine serum albumin (BSA) as a standard.

### Total RNA extraction and cDNA synthesis

Total RNA was extracted from 4 × 10^6^ purified merozoites using a QIAamp^®^ RNA blood mini kit (Qiagen, Hilden, Germany). The RNA preparations were additionally treated with RNase-Free DNase (Qiagen) for 15 min at room temperature according to the manufacturer’s instructions to remove any traces of DNA. Total RNA was quantified using a NanoDrop^®^ 2000 (Thermo Fischer Scientific, Waltham, MA, USA) and the integrity was assessed by electrophoresis on a 1% agarose gel containing ethidium bromide. cDNA was then synthesized from the total RNA using an iScript™ cDNA synthesis kit (BioRad, Hercules, CA, USA).

### Molecular cloning of CSUI_005805 full length cDNA

The complete coding region of gene CSUI_005805 (1170 bp) (Additional file 1) was obtained by polymerase chain reaction (PCR) amplification using gene-specific primers (forward: 5′-c***GA GCT C***AA TAC GTC CGG CGT GAA AAT GT-3′; reverse: 5′-gc***G TCG AC***C TAT AGG AGT TCC ACT AAG GTT-3′). Unique restriction endonuclease recognition sites (bold and italics) were included at the 5′-termini of the primers to facilitate the directional cloning. The target sequence was amplified under the following conditions: an initial denaturation step at 95 °C for 5 min; followed by 40 cycles of 94 °C for 15 s, 64 °C for 1 min, 72 °C for 1.5 min and a final elongation step at 72 °C for 10 min. Amplification products were loaded onto a 1% agarose gel stained with ethidium bromide to determine the size of the amplified products.

The amplicons were gel purified using a QIAquick^®^ gel extraction kit (Qiagen), ligated into the pDrive^®^ cloning vector (Qiagen), and then used to transform competent Qiagen EZ *Escherichia coli* cells (Qiagen). The resultant transformants were selected on a Luria-Bertani agar plate supplemented with 100 μg/ml ampicillin (Sigma-Aldrich, St Louis, MO, USA) for resistance and 50 μM 5-bromo-4-chloro-3-indolyl-β-D-galactopyranoside (Sigma) and 80 μg/ml isopropyl-1-thio-β-D-galactopyranoside (IPTG) (Sigma) for blue/white screening. Six recombinant (white) clones were selected and tested by colony PCR using vector-specific primers. To confirm the integrity of the coding sequences, recombinant plasmids pDRIVE-CSUI_005805 were purified using QIAprep^®^ spin miniprep kit (Qiagen) and sequenced (Microsynth Austria GmbH, Vienna, Austria) using vector-specific primers.

### Sequence analysis of CSUI_005805

The sequences obtained for the CSUI_005805 cDNA were analyzed for similarity using BLAST programs at NCBI (http://blast.ncbi.nlm.nih.gov/Blast.cgi) and ToxoDB (http://toxodb.org/toxo/showQuestion.do?questionFullName=UniversalQuestions.UnifiedBlast). The deduced amino acid sequence was obtained using the open reading frame (ORF) finder at NCBI (https://www.ncbi.nlm.nih.gov/orffinder/). The molecular mass, instability index and theoretical isoelectric point were obtained using the ProtParam tool of the ExPASy server of the Swiss Institute of Bioinformatics (http://web.expasy.org/protparam/). Signal peptides, transmembrane regions, subcellular localization and protein motifs were predicted using SignalP (http://www.cbs.dtu.dk/services/SignalP/), Phobius (http://phobius.sbc.su.se/), PredictProtein (https://www.predictprotein.org/) and Motifscan (http://myhits.isb-sib.ch/cgi-bin/motif_scan) computational tools, respectively.

### CSUI_005805 transcription at different time-points of *C. suis* development in vitro

Real-time quantitative PCR (qPCR) was used to quantify CSUI_005805 transcripts at different time-points of the *C. suis* development in vitro, namely in free sporozoites released from sporulated oocysts, intracellular merozoites on days 1, 3 and 6 p.i. and extracellular merozoites released into the medium on days 5–6 p.i. Total RNA was extracted from parasites or infected cell cultures at each time-point using an RNeasy^®^ mini kit (Qiagen) and treated with RNase-free DNase (Qiagen) to remove any DNA contamination. First-strand cDNA templates were synthesized from 1 μg of total RNA using an iScript™ cDNA synthesis kit as described above. Quantitative PCR amplification of cDNA from each time-point of in vitro development was carried out in a Mx3000P thermal cycler (Agilent Technologies, Santa Clara, CA, USA) employing forward and reverse primers (200 nM each; see Table 1 for details) and 1× SsoAdvanced™ universal SYBR^®^ Green supermix (BioRad, Hercules, CA, USA) in a total volume of 20 μl using following cycling conditions: initial separation of DNA strands at 95 °C for 30 s, followed by 40 cycles of 95 °C for 15 s, 60 °C for 30 s, and 72 °C for 30 s, and one cycle of 95 °C for 30 s, 60 °C of 30 s, and 95 °C for 30 s for melting-curve analysis. Each sample was run in triplicate and the complete experiment was performed in two separate biological replicates. The qPCR results were normalized against each of the four reference genes, namely glyceraldehyde-3-phosphate, actin, 18S ribosomal RNA and large subunit ribosomal RNA genes. Average gene expression relative to the endogenous control for each sample was calculated using the 2^−ΔΔCt^ method described by Livak & Schmittgen [30]. Primers for CSUI_005805 gene and four reference genes (Table 1) were designed using Primer3Plus software (http://www.bioinformatics.nl/cgi-bin/primer3plus/primer3plus.cgi/).

**Table 1:** Oligonucleotide primers used for qPCR to determine stage-dependent transcription of CSUI_005805

Gene name	Forward primer sequence 5′-3′	Reverse primer sequence 5′- 3′	Amplicon length (bp)
GAPDH	ATTGGTCGTCTCGTGTTCCG	GATCGCACTTGGCTCCTTCT	216
ACT	CTTGCTGGCCGTGATTTGAC	ATATTGCCGTCCGGAAGCTC	203
18S rRNA	GCCAGTAGTCATATGCTTGTC	CTAATAAACACTGCCCTTCCTG	195
LSU rRNA	TGATTCCGAAGAGTGAGGC	CCAGGCGAAACTATAAAGCAG	195
CSUI_005805	CCTGAAAGTCGCCTGTCCAT	GACGCGTCAGCCGTTATAGT	224

*GAPDH* Glyceraldehyde-3-phosphate, *ACT* Actin, *18S rRNA* 18S ribosomal RNA, *LSU rRNA* Large subunit ribosomal RNA

### Expression and purification of the recombinant CSUI_005805 protein

The plasmid pDRIVE-CSUI_005805 with correct sequence and orientation was digested with the restriction enzymes *SacI* and *SalI*. The target fragment was then purified, ligated into the expression vector pQE-31 (Qiagen) digested by the same restriction enzymes, and used to transform competent *E. coli* M_15_ pREP_4_ cells (Qiagen) for protein expression. Appropriate target and correct orientation of the inserts were confirmed by colony PCR, restriction analysis and sequencing using pQE vector specific primers. The recombinant protein expression by *E. coli* clones was induced by adding IPTG (final concentration 1 mM) after the OD_600_ of the culture reached 0.6. The induced bacterial cells were incubated at 37 °C for 1 h after adding IPTG and then harvested by centrifugation. Cell pellets were lysed in lysis buffer (100 mM NaH_2_PO_4_, 10 mM Tris-Cl, 8 M urea, pH 8.0) containing ProteoGuard™ EDTA-Free Protease Inhibitor Cocktail (Clontech, Mountain View, CA, USA) followed by sonication (65%, 60 s) and incubation under constant vortexing for 30 min at room temperature. Next, the lysate was separated by SDS-PAGE on a 12% gel and visualized after staining with silver nitrate. The recombinant (His)_6_ tagged CSUI_005805 proteins (rCSUI_005805) were purified from the soluble fraction of lysates by Ni^2+^-nitrilotriacetic acid (Ni-NTA) chromatography [NiNTA spin columns (Qiagen) for small expression culture and NiNTA agarose (Qiagen) for standard expression culture], as described in the protocols outlined in the QIA *expressionist* handbook for high-level expression and purification of (His)_6_ tagged proteins (https://www.qiagen.com/at/resources/resourcedetail?id=79ca2f7d-42fe-4d62-8676-4cfa948c9435&lang=en; June 2003). Protein purity was visualized on a 12% SDS-PAGE gel by silver staining and the concentration was measured by a Bradford assay as described before. The purified protein was stored in aliquots at -20 °C until used.

### Mass spectrometry

The protein bands corresponding to rCSUI_005805 were manually excised from silver stained SDS-PAGE gels and subsequently digested *in-gel* using trypsin (Trypsin Gold, Mass Spectrometry Grade, Mannheim, Germany). The extracted peptides were then subjected to protein identification and quantification using a high-resolution quadrupole time of flight mass spectrometer (Triple TOF 5600+, AB Sciex, Foster City, CA, USA) coupled to a nano-HPLC Ultimate 3000 RSLC system (Dionex, Amsterdam, The Netherlands). The processed MS spectra were searched against UniProt DB (downloaded from the publicly available UniProt server (www.uniprot.org)) together with the in-house generated *C. suis* database.

### Anti-rCSUI_005805 monospecific polyclonal serum production

Seven-week-old specific pathogen-free (SPF) white leghorn chicken (*n* = 10) were immunized intramuscularly three times at 2-week intervals with purified rCSUI_005805. Primary immunization was performed with 0.1 mg of purified rCSUI_005805 in Freund’s complete adjuvant (Sigma-Aldrich) as a 1:1 emulsion. The birds were boostered twice with the same amount of purified rCSUI_005805 in Freund’s incomplete adjuvant (Sigma-Aldrich). Two weeks after the final booster immunization, birds were bled for collection of serum (chicken anti-rCSUI_005805 polyclonal sera). Sera collected before immunization was used as negative control sera (pre-immune chicken sera).

### Immunoblot analysis of rCSUI_005805 and crude merozoite lysate

Crude protein lysate from purified merozoites and rCSUI_005805 was subjected to SDS-PAGE on a 12% gel and the resolved protein bands were visualized using silver nitrate. The protein bands from the unstained gels were transferred to nitrocellulose (NC) membranes (BioRad) for immunoblot. After blocking for 1 h with 2% BSA in PBS at room temperature, the NC membranes were incubated with anti-His horseradish peroxidase (HRP) conjugates (Qiagen), chicken anti-rCSUI_005805 polyclonal sera (dilution, 1:200), highly positive porcine anti-*C. suis* polyclonal sera from experimentally infected piglets (dilution, 1:200) [31, 32] or the negative chicken sera (dilution, 1:200) diluted in TTBS buffer (100 mM Tris, 0.9% NaCl, 0.1% Tween 20) for 30 min at room temperature. To test cross-reactivity of naïve and recombinant antigenic proteins of *C. suis* to *Eimeria* spp., the NC membranes were incubated separately with field sera obtained from chickens that had been vaccinated with HIPRACOX^®^ (HIPRA, Amer, Spain). After rinsing in TTBS for 15 min, blots were exposed to biotinylated goat anti-pig IgG (dilution, 1:400) or biotinylated goat anti-chicken IgY (dilution, 1:300) (Vector Laboratories, Burlingame, CA, USA) diluted in TTBS buffer as secondary antibody for 30 min at room temperature, incubated with avidin-biotin complex solution (Vector Laboratories) and finally detected by 3,3′-5,5′-tetramethylbenzidine, according to the manufacturer’s instructions.

### Immunofluorescence analysis

Purified extracellular merozoites and sporozoites were transferred to poly-L-lysine treated glass slides (Polysciences Inc., Hirschberg an der Bergstrasse, Germany) and air dried before fixation. Parasites were either fixed with 4% paraformaldehyde in PBS for 10 min followed by permeabilization with 0.25% TritonX-100 in PBS for 10 min or fixed in ice-cold 100% methanol for 10 min and then blocked with 4% BSA in PBS for 2 h at room temperature. A 1:50 dilution of chicken anti-rCSUI_005805 polyclonal sera was added and incubated for 2 h at room temperature followed by 1 h incubation with a 1:300 dilution of Alexa Fluor^®^ (A488) goat anti-chicken IgG (Invitrogen, Eugene, OR, USA). Nuclei were visualized by staining with 1 μg/ml of 4,6-diamidino-2-phenylindole (DAPI) (Sigma-Aldrich) for 5 min prior to mounting under coverslips with Aqua-Poly/Mount (Polysciences Inc.). The slides were washed five times with PBS for 25 min after each step described above. Imaging was done on a Zeiss Axio Imager Z2 wide-field fluorescence microscope (×63 oil immersion objective) and a Zeiss LSM 510 Meta-confocal laser scanning microscope (×63 oil immersion objective). Images were analyzed with Light Editions of Zen 2012 and 2009 (Carl Zeiss Microimaging GmbH, Jena, Germany).

### Inhibition of host-cell invasion in vitro

A qPCR assay was developed in combination with in vitro culture to determine efficacy of the inhibition of host-cell invasion by sporozoites. IPEC-J1 cells (1.5 × 10^5^ cells/well) were seeded in 12-well plates (TPP, Trasadingen, Switzerland) and cultured in Dulbecco’s Modified Eagle’s Medium (DMEM-Ham’s F12) (Gibco, Grand Island, NY, USA) supplemented with 5% fetal calf serum, 2 mM L-glutamine, 100 U/ml penicillin and 0.1 mg/ml streptomycin (Gibco) at 37 °C with 5% CO_2_ for 24 h. Freshly-excysted sporozoites were counted and pre-incubated at 37 °C with different concentrations of either chicken anti-rCSUI_005805 or porcine anti-*C. suis* polyclonal sera for 2 h. The corresponding concentrations of pre-immune chicken serum or previously collected pre-colostral piglet serum [31, 32] were used as a negative controls and equivalent volumes of complete DMEM-Ham’s F12 medium as a baseline control. Pre-incubated sporozoites (2 × 10^3^/well) were used to infect IPEC-J1 cells and the cells were allowed to grow at 40 °C with 5% CO_2_ for 24 h. Cultures were then washed four times with PBS and subjected to DNA extraction using a peqGOLD Microspin Tissue DNA Kit (peqlab, Erlangen, Germany), according to the manufacturer’s instructions. The DNA was eluted with 75 μl of elution buffer and subjected to qPCR for *C. suis* genome quantification using the large subunit rRNA gene (LSU rRNA gene) as target (GenBank accession number: AF093428.1). qPCR was performed in a Mx3000P thermal cycler (Agilent Technologies, Santa Clara, CA, USA) using 400 nM of each primer (forward: 5′-TGA TTC CGA AGA GTG AGG C -3′; reverse: 5′-CCA GGC GAA ACT ATA AAG CAG -3′), 200 nM probe (5′- FAM-TCC GGC ATT GAT CCC TCT GCT TTA TCC C-BHQ1-3′) and 5 μl undiluted template DNA with SsoAdvanced™ Universal Probes Supermix (BioRad). Each sample was run in triplicate and the experiment was performed twice under the following cycling conditions: 95 °C for 10 min followed by 40 cycles with 95 °C for 30 s and 60 °C for 1 min. In vitro inhibition percentage for each culture was calculated as follows:

$$ \%\ \mathrm{o}\mathrm{f}\ \mathrm{in}\mathrm{hibition}=100 \times \left(1\ \hbox{-} \frac{\mathrm{average}\ \mathrm{n}\mathrm{o}.\ \mathrm{o}\mathrm{f}\  C. suis\ \mathrm{gene}\ \mathrm{copies}\ \mathrm{in}\ \mathrm{serum}\ \mathrm{treated}\ \mathrm{cultures}}{\begin{array}{c}\hfill \mathrm{average}\ \mathrm{n}\mathrm{o}.\ \mathrm{o}\mathrm{f}\  C. suis\ \mathrm{gene}\ \mathrm{copies}\ \mathrm{in}\ \mathrm{n}\mathrm{o}\mathrm{n}\hbox{-} \mathrm{treated}\ \mathrm{cultures}\ \hfill \\ {}\hfill \left(\mathrm{baseline}\ \mathrm{control}\right)\hfill \end{array}}\right) $$

The differences among experimental groups were tested by one-way ANOVA using Microsoft Excel 2007, with significance reported at *P* < 0.05.

## Results

### Cloning and sequence analysis

**Fig. 1:**
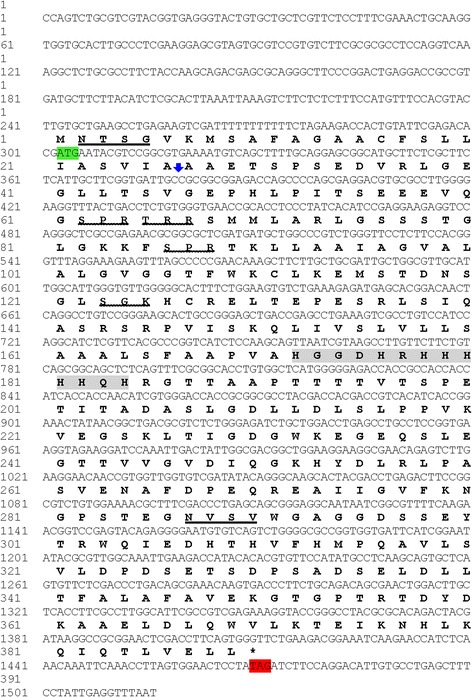
The nucleotide sequence and deduced amino acid sequence of complete coding region of CSUI_005805 gene. *Vertical arrow*, signal peptide cleavage site; *green shaded*, start codon; *red shaded*, stop codon; *wavy underlined*, putative protein kinase C phosphorylation sites; *underlined*, putative N-glycosylation site; *gray shaded*, histidine rich region

**Fig. 2:**
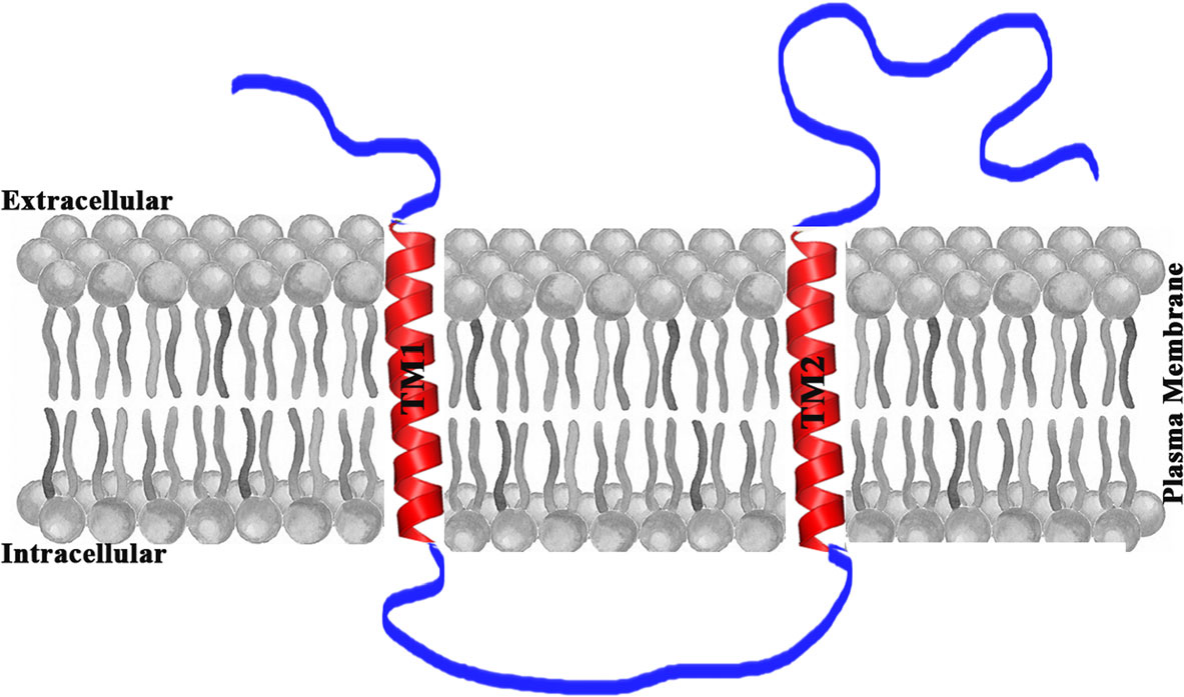
Schematic representation of predicted transmembrane topology of CSUI_005805 protein. It consists of two transmembrane domains (TM) with extracellular N and C termini and a long cytoplasmic loop extending between TM1 andTM2

The CSUI_005805 gene included a 302 bp 5′-untranslated region (5′-UTR) before the ATG initiation codon and a 1170 bp coding sequence terminating with the TAG stop codon (Fig. 1), followed by a 44 bp 3′-UTR. A single 1170 bp CSUI_005805 ORF encoded a protein of 389 amino acids with the predicted molecular mass of 42 kDa. The theoretical isoelectric point and instability index were 5.52 and 47.77, respectively. The deduced amino acid sequence had a predicted N-terminal 19-amino acids signal peptide (1–26) for entrance into the secretory pathway, and it also had two predicted transmembrane domains (TM1:91–109; TM2:152–171) in the C-terminal region of the protein that could serve as membrane anchorage (Fig. 2). However, it had no identifiable homology to known proteins that might allude to its function. Motifscan predicted two putative glycosylation sites and four putative protein kinase C phosphorylation sites (Fig. 1).

### Real-time quantitative PCR analysis of CSUI_005805 transcripts

**Fig. 3:**
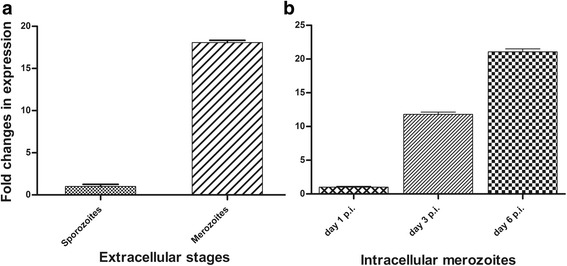
Relative mRNA expression levels of CSUI_005805 in vitro. **a** Free sporozoites and merozoites. **b** Intracellular merozoites on day 1, 3 and 6 post-infection (p.i.). The data are displayed as mean ± standard deviation

The expression profile of the CSUI_005805 mRNA was examined at different time-points of *C. suis* development in vitro using qPCR. Among the extracellular stages, CSUI_005805 transcripts in merozoites were 18-fold higher (Fig. 3a) compared to sporozoites, which agrees with the high expression level in merozoites measured from an RNA-Seq experiment [27]. Additionally, the level of CSUI_005805 transcripts on day 3 and 6 p.i. were 12- and 21-fold higher, respectively, in intracellular merozoites compared to that of day 1 p.i. (Fig. 3b).

### Expression and purification of rCSUI_005805

**Fig. 4:**
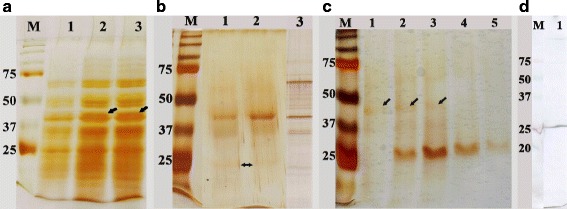
Expression of rCSUI_005805. Lane M: molecular weight marker. **a** Analysis of expressed recombinant protein by SDS-PAGE: uninduced culture (Lane 1); induced culture with 1 mM IPTG for 0.5 h (Lane 2) and 1 h (Lane 3), *arrows* indicate target protein bands of ~42 kDa. **b** SDS-PAGE analysis of NiNTA spin column purified rCSUI_005805: elution 1 (Lane 1); elution 2 (Lane 2), *arrows* indicate faint protein bands of ~25 kDa; naïve merozoite lysate (Lane 3). **c** Batch purified rCSUI_005805 using NiNTA agarose: stepwise elution (Lanes 1–5), *arrows* indicate faint protein bands of ~42 kDa. **d** Immunoblot of batch purified rCSUI_005805 probed with anti-His HRP conjugate (Lane 1)

The recombinant CSUI_005805 protein (rCSUI_005805) was expressed in M15 pREP4 *E. coli* as a His-tagged fusion protein. A PCR product of 1170 bp was purified from an agarose gel and cloned into a pre-linearized pQE-31 bacterial expression vector containing N-terminal (His)_6_ tag prior to transformation of chemically competent *E. coli* M15 pREP4 cells. rCSUI_005805 expressed in bacterial lysate revealed a major protein band of ~42 kDa after induction with 1 mM IPTG for 1 h at 37 °C indicating that this was the most appropriate incubation time for expression (Fig. 4a, Lane 3). A band of the target protein was not detected in the bacterial culture that had not been induced with IPTG (Fig. 4a, Lane 1). To isolate any tagged proteins independent of their solubility and location within the cells, purification of rCSUI_005805 was performed under denaturing conditions which allows solubilization of most proteins and inclusion bodies [33], thereby facilitating their direct analysis by SDS-PAGE.

Purification of small expression cultures (10 ml) with NiNTA spin columns after induction with IPTG showed a major protein band of ~ 42 kDa together with a smaller protein band of ~ 25 kDa (Fig. 4b, Lanes 1, 2). However, when the expression was scaled up to 500 ml and was batch purified using NiNTA agarose with step-wise elution in elution buffer (100 mM NaH_2_PO_4_, 10 mM Tris-Cl, 8 M urea, pH 4.5), SDS-PAGE showed a major protein band of ~ 25 kDa and only a faint band of ~ 42 kDa (Fig. 4C, Lanes 1–5). LC-MS/MS analysis of the 25 kDa band corroborated the presence of a part of the CSUI_005805-coded protein as deduced by nucleic acid translation. Additionally, as (His)_6_ tagged protein, the expression and characterization of the rCSUI_005805 was verified by immunoblot analysis with an anti-His HRP conjugate (Qiagen), reacting specifically as expected (Fig. 4d).

### Immunoblot analysis of rCSUI_005805 and crude merozoite lysate

**Fig. 5:**
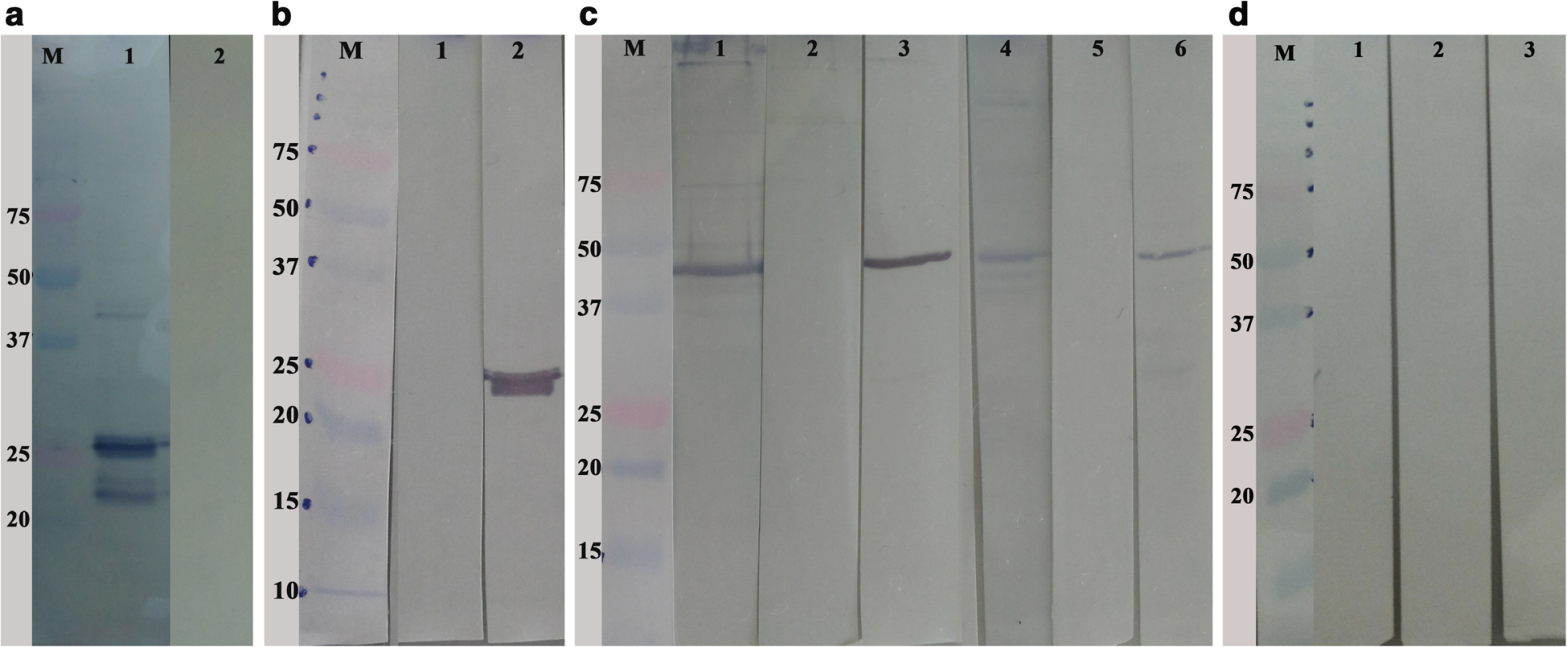
Immunoblot analyses of rCSUI_005805 and naïve merozoite lysate. The expressed recombinant protein and crude merozoite lysate were separated by SDS-PAGE and transferred to NC membranes. Lane M: Molecular weight marker (ordinate values describe band sizes in kDa); **a** Immunoblot of rCSUI_005805 probed with porcine anti-*C. suis* polyclonal sera (Lane 1) and with porcine pre-colostral sera (Lane 2). **b** Immunoblot of rCSUI_005805 probed with negative chicken serum (Lane 1) and chicken anti-rCSUI_005805 polyclonal sera (Lane 2). **c** Immunoblot of insoluble (Lanes 1–3) and soluble fractions (Lanes 4–6) of naïve merozoite lysate probed with porcine anti-*C. suis* polyclonal sera (Lanes 1 and 4), chicken anti-rCSUI_005805 polyclonal sera (Lanes 3 and 6) and negative chicken sera (Lanes 2 and 5), respectively. **d** Immunoblot of naïve merozoite lysate (Lanes 1 and 2) and rCSUI_005805 (Lane 3) probed with field sera from chickens vaccinated with HIPRACOX^®^

The immunoblot of purified rCSUI_005805 probed with highly positive porcine anti-*C. suis* polyclonal serum could detect the expected ~ 25 kDa and ~ 42 kDa protein bands indicating that bacterially expressed rCSUI_005805 maintained the antigenic determinants recognized by protective porcine antibodies compared to their naïve protein counterparts (Fig. 5a, Lane 1). Apart from these two bands, a lower molecular weight protein band (~23 kDa) was also detected by porcine anti-*C. suis* polyclonal sera, which might represent either (a) degraded products, (b) N-terminal proteolytic truncations of rCSUI_005805, since it was only detected by porcine anti-*C. suis* antibodies but not by anti-His HRP conjugates that recognize the N-terminal poly-histidine tag on recombinant proteins or (c) an unrelated antigenic protein co-purified together with rCSUI_005805. These bands were not detected when porcine pre-colostral serum was used (Fig. 5a, Lane 2). Similarly, chicken anti-rCSUI_005805 polyclonal sera also recognized purified rCSUI_005805 (~25 kDa) by immunoblotting (Fig. 5b, Lane 2), which confirmed the specificity of the polyclonal sera produced. No bands were detected when the immunoblots were probed with negative (pre-immune) chicken sera (Fig. 5b, Lane 1). Furthermore, to confirm that the chicken anti-rCSUI_005805 sera did in fact recognize the native form of CSUI_005805 protein and not a cross-reactive epitope, an analysis was carried out in which crude merozoite extract was probed with anti-rCSUI_005805 serum. Naïve protein of 42 kDa in insoluble (Lanes 1–3) and soluble (Lanes 4–6) fractions of crude merozoite extract were recognized by chicken anti-rCSUI_005805 polyclonal sera (Fig. 5c, Lanes 3, 6) in a pattern like those identified by highly positive porcine anti-*C. suis* polyclonal sera (Fig. 5c, Lanes 1, 4). As expected, negative chicken serum also failed to detect any bands in an immunoblot of naïve merozoite proteins (Fig. 5c, Lanes 2, 5). None of the antigenic proteins (naïve or rCSUI_005805) of *C. suis* were recognized by serum antibodies from chickens vaccinated with HIPRACOX^®^ in an immunoblot (Fig. 5d), indicating that *C. suis* does not share common epitopes with avian *Eimeria* spp. that are detectable by an immunoblot analysis.

### Immunolocalization of CSUI_005805 antigens in merozoites and sporozoites

**Fig. 6:**
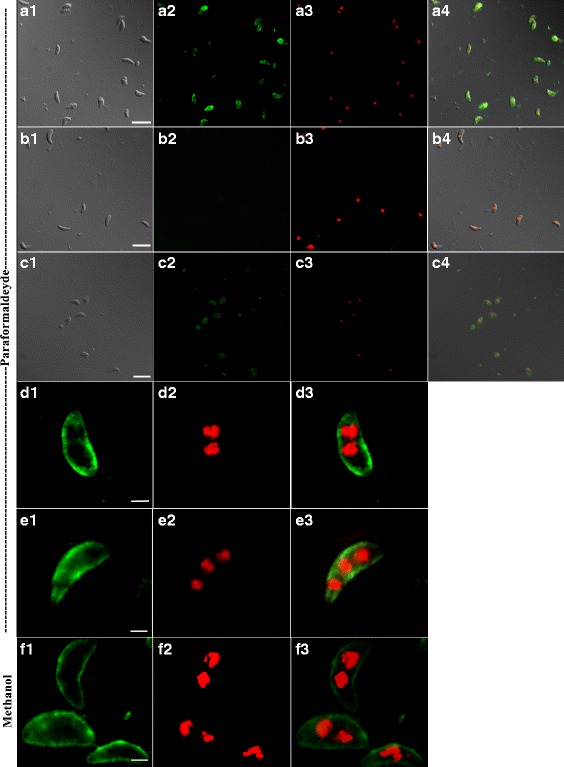
Localization of CSUI_005805 antigens in merozoites and sporozoites. **a**–**c** Wide-field epifluorescence microscope (×63 magnification; *scale-bar*: 10 μm). **d**–**f** Meta confocal laser scanning microscope (×63 magnification; *scale-bar*: 5 μm). **a** *C. suis* merozoites: (**a1**) Differential interference contrast (DIC); (**a2**) Localization using A488; (**a3**) Nuclear staining with DAPI (**a4**) DIC, A488 and DAPI merged. **b** Negative control, merozoites probed with negative chicken serum: (**b1**) DIC; (**b2**) A488; (**b3**) DAPI; (b4) merged. **c** *C. suis* sporozoites: (**c1**) DIC; (**c2**) A488; (**c3**) DAPI; (**c4**) merged. **d** and **e** Paraformaldehyde-fixed *C. suis* merozoites: (**d1**, **e1**) A488; (**d2**, **e2**) DAPI; (**d3**, **e3**) merged. **f** Methanol-fixed *C. suis* merozoites: (**f1**) A488; (**f2**) DAPI; (**f3**) merged

Fixed extracellular merozoites and sporozoites were immune-stained with chicken anti-rCSUI_005805 polyclonal serum, visualized with A488 (green) and counter-stained with DAPI (red). Immunofluorescence based localization studies in merozoites showed that irrespective of the fixation techniques used, the labelled antigen was mainly concentrated towards the surface of merozoites (Fig. 6). In some cases, staining was observed towards surface, except for a small gap at the extreme apical and basal ends of merozoites (Fig. 6e), indicating apparent localization of rCSUI_005805 with the inner membrane complex (IMC) [34]. Moreover, the presence of signal peptide and transmembrane domains suggests that rCSUI_005805 might be synthesized as mature protein in the cytosol and then transported to the external membrane. The intensity of A488 fluorescence was much lower in fixed extracellular sporozoites than that of merozoites (Fig. 6c). No reactivity was observed when merozoites were stained with negative chicken serum as a primary antibody (Fig. 6b).

### Serum invasion-inhibition assay

Porcine anti-*C. suis* and chicken anti-rCSUI_005805 polyclonal sera were tested for their ability to inhibit the invasion of cultured IPEC-J1 cells in vitro by *C. suis* sporozoites. The highest serum concentration that could be employed was 2.5% because higher concentrations lead to non-specific and non-reproducible results in the assay (details not shown). Pre-treatment with positive serum antibodies decreased the invasion capacity of sporozoites as determined by evaluating the number of parasite stages in culture 24 h after infection, and the observed inhibition effect was dose-dependent. Positive chicken anti- rCSUI_005805 serum (2.5%) decreased the number of intracellular stages by 28.3% compared to the baseline control and by 50.6% compared to the pre-immune serum of the same concentration. In comparison, 2.5% positive piglet serum decreased the detected number of intracellular stages by 62.1% compared to the baseline and by 74.2% compared to pre-colostral serum of the same concentration. Similarly, 1.25% positive piglet and chicken sera decreased the number of detected stages by 18.5 and 6.8%, respectively, compared to the baseline control and 46.2 and 15.9%, compared to the respective pre-immune serum. For both chicken and piglet sera, pretreatment with 2.5 or 1.25% positive sera significantly decreased (*F*_(1,10)_ = 13.15, *P* = 0.004; *F*_(1,10)_ = 29.42, *P* = 0.0002, respectively) the invasion capacity of sporozoites while other concentrations had no inhibiting effects.

## Discussion

*Cystoisospora suis* is an intracellular apicomplexan parasite that develops entirely in single host [35]. Like in other coccidia, replicating merozoites and gamonts are the most pathogenic stages [32, 36, 37], leading to host cell destruction and thus altering intestinal permeability. The global distribution of cystoisosporosis with high prevalence and limited control options demands innovation strategies for novel alternative control measures. The role of humoral immune responses in terms of conferring protective immunity to coccidial infection is debatable. However, evidences of protection by parasite-specific antibodies, either maternally derived or self-produced, have been reported in *Eimeria* [38–41] and recently in *C. suis* [32]. Protective antibodies might thus be important for the defense against invasive stages, sporozoites and merozoites. *Cystoisospora suis* merozoites express between 9000 and 10,000 proteins during their development [27], thereby exposing the host to a complex assembly of antigens. Logically, the most reliable and effective antigens for raising protective immunity against *C. suis* would be those that are parasite-specific, highly expressed and associated with invasion and/or progression of pathogenesis in the host.

Proteins of unknown function comprise 30–40% of the total number of proteins annotated from any genome [42]. In all sequenced coccidian genomes, a large fraction of the predicted genes encodes for proteins with no orthologs and/or unknown function [27, 43–46]. These could constitute a rich reservoir for species-specific antigens and thus putative targets for immune-intervention. In line with this trend, previous screening of the predicted proteome of *C. suis* with the software Vacceed revealed numerous potential vaccine candidates with no annotated function [27]. Among these, CSUI_005805, which encodes for an uncharacterized protein, appeared to be an attractive candidate owing to its species-specificity and high expression in merozoites. In the present study, the entire coding sequence of the CSUI_005805 gene was cloned and characterized. A single CSUI_005805 ORF of 1170 bp encoded for 389 amino acids with a predicted molecular mass of 42 kDa and contained a predicted signal peptide and two predicted transmembrane domains. The presence of signal peptide and transmembrane domains suggest that the protein enters the secretory pathway and is actively transported out of the cell to anyone of the numerous cellular membrane systems [47]. Signal peptides are found both in secreted and most cell surface proteins [48], but proteins with predicted signal peptide in combination with transmembrane domains are considered membrane-spanning [47] and might escape secretion. Thus, it is likely that CSUI_005805 is a secretory protein with a signal peptide and/or one or more transmembrane domains for membrane anchorage [49]. Previous studies suggested that most transmembrane proteins encoded by apicomplexans localize to the external membrane or IMC rather than organellar membranes [50–52]. Also, it has recently been reported that in *Plasmodium* components of the IMC are surface-exposed and accessible to antibodies [53], and presumably this might also be the case in *C. suis*, indicating that these proteins could be good targets when selecting for vaccine candidates.

The gene encoding the complete CSUI_005805 protein was cloned into the bacterial expression vector pQE-31 to express the recombinant protein rCSUI_005805 in a size similar to the predicted molecular mass that was present in extracts of the *E. coli* host as visualized in SDS-PAGE by silver staining. However, batch purification of rCSUI_005805 using NiNTA agarose resulted in a faint 42 kDa and a major 25 kDa protein band, suggesting that rCSUI_005805 either undergoes proteolytic maturation yielding smaller 25 kDa protein fragments or the smaller protein band might be a breakdown product of rCSUI_005805 during affinity purification. Additionally, LC-MS/MS analysis of this smaller protein band was consistent with the amino acid sequence predicted for the CSUI_005805 protein. The observed unstable nature of rCSUI_005805 is also in agreement with the predicted instability index value of 47.77 as proteins with an index > 40 are considered unstable [54].

During expression and purification, rCSUI_005805 maintained a conformation that was recognized by antiserum raised against naïve proteins in immunoblotting, indicating that despite bacterial expression, rCSUI_005805 retained antigenic and immunogenic properties and could induce an antibody response in the host. Chicken anti-rCSUI_005805 antibodies could identify soluble and insoluble fractions of naïve merozoite proteins in a pattern similar to that identified by sera of experimentally infected piglets, further suggesting that rCSUI_005805 mimics the antigenic properties elicited by immunization with naïve antigens.

Identification of genes expressed in various stages of the life-cycle of a parasite is critical to understand its developmental biology [55–57] and immunological “bottlenecks” where it is most vulnerable. Relative expression patterns of CSUI_005805 mRNA levels at different cultured stages of *C. suis* showed that CSUI_005805 was differentially expressed with higher transcription in merozoites compared to sporozoites. Once the sporozoites invaded the enterocytes, the transcription level increased steadily throughout the entire merogony, indicating that this protein is important for the survival and establishment of merozoites. Expression of CSUI_005805 transcripts in other stages, especially gamonts, should be investigated to complete the picture.

Chicken anti-rCSUI_005805 polyclonal serum was used to determine the subcellular localization of the CSUI_005805 protein in fixed extracellular sporozoites and merozoites. Apart from a slightly diffuse cytoplasmic distribution, a major proportion localized at the surface of the parasite, indicating that the investigated protein might be secreted and then translocated to the external membrane as indicated by the results of *in silico* analyses outlined above. Fixation techniques with either paraformaldehyde or methanol did not alter the localization pattern of this protein. In sporozoites fluorescence intensity of A488 was lower compared to merozoites with both fixation techniques, which supports the results of qPCR that detected increasing levels of CSUI_005805 transcription during in vitro cultivation of *C. suis* as described above.

The ability of apicomplexan parasites to invade host cells is key to their survival and pathogenesis [58]. Host cell invasion is a complex process and is relatively conserved among the members of this taxon [59]. Genomic DNA extracted from parasites after cultivation for 24 h can be used as a template for quantitatively assessing invasion [60]. Sporozoites pre-incubated with 2.5% anti-rCSUI_005805 antibodies prior to infection of IPEC-J1 cells slightly reduced the number of intracellular parasites whereas inhibition of host-cell invasion by porcine anti-*C. suis* serum antibodies in the same dilution was two-fold higher, indicating that CSUI_005805 does not play a leading role in the invasion of the host-cell, at least not by itself.

## Conclusions

We successfully cloned and characterized a novel gene of *C. suis*, CSUI_005805, which encodes for a species-specific, differentially expressed transmembrane protein. The recombinant CSUI_005805 protein seemed to undergo proteolytic maturation after purification but retained the antigenic and immunogenic properties and is recognized by host antibodies. In vitro expression of CSUI_005805 transcripts increases steadily as soon as sporozoites invade IPEC-J1 cells in vitro, indicating that it might be crucial for establishment and/or growth of merozoites inside the host cells. Currently, no genetic manipulation technique to confirm the direct implication of CSUI_005805 in parasite development is available for *C. suis*. Nevertheless, we have shown that rCSUI_005805 could explicitly induce an antibody response in infected hosts, and given that humoral immune responses confer at least partial protection in *C. suis*, this protein could be exploited for the development of alternative control strategies, possibly in combination with other complementary antigens.

## Additional file

Additional file 1: Complete coding DNA sequence (CDS) of the CSUI_005805 gene. (DOCX 13 kb)

## Abbreviations

(His)_6_: 6× histidine
A488: Alexa fluor 488
ACT: Actin
BSA: Bovine serum albumin
DAPI: 4, 6-diamidino-2-phenylindole
DMEM: Dulbecco’s modified eagle’s medium
GAPDH: Glyceraldehyde-3-phosphate
HRP: Horseradish peroxidase
IgY: Immunoglobulin Y
IMC: Inner membrane complex
IPEC: Intestinal porcine epithelial cells
IPTG: Isopropyl-1-thio-β-D-galactopyranoside
LC-MS: Liquid chromatography-mass spectrometry
LSU rRNA: Large subunit ribosomal RNA
NiNTA: Ni^2+^-nitrilotriacetic acid
OD: Optical density
PBS: Phosphate buffer saline
PCR: Polymerase chain reaction
qPCR: Quantitative real-time polymerase chain reaction
rRNA: Ribosomal RNA
SDS: Sodium dodecyl sulfate
SPF: Specific pathogen free
TM: Transmembrane domain
TTBS: Tris-buffered saline
UTR: Untranslated region
