# Supplementary material for: Cloning, expression and molecular characterization of a Cystoisospora suis specific uncharacterized merozoite protein
Source: Parasit Vectors. 2017 Feb 7;10:68. doi: 10.1186/s13071-017-2003-1 (PMC5297187; doi:10.1186/s13071-017-2003-1)
Supplement: Additional file 1: — Complete coding DNA sequence (CDS) of the CSUI_005805 gene. (DOCX 13 kb) [file 13071_2017_2003_MOESM1_ESM.docx]

>CSUI_005805 organism=*Cystoisospora_suis*, sequence_type = CDS

ATGAATACGTCCGGCGTGAAAATGTCAGCTTTTGCAGGAGCGGCATGCTTCTCGCTTCTCATTGCTTCGGTGATTGCCGCGGCGGAGACCAGCCCCAGCGAGGACGTGCGCCTTGGGGAAGGTTTACTGACCTCTGTGGGTGAACCGCACCTCCCTATCACATCCGAGGAAGAGGTCCAGGGCTCGCCGAGAACGCGGCGCTCGATGATGCTGGCCCGTCTGGGTTCCTCTTCCACGGGTTTAGGAAAGAAGTTTAGCCCCCGAACAAAGCTTCTTGCTGCGATTGCTGGCGTTGCATTGGCATTGGGTGTTGGGGGCACTTTCTGGAAGTGTCTGAAAGAGATGAGCACGGACAACTCAGGCCTGTCCGGGAAGCACTGCCGGGAGCTGACCGAGCCTGAAAGTCGCCTGTCCATCCAGGCATCTCGTTCACGCCCGGTCATCTCCAAGCAGTTAATCGTAAGCCTTGTTCTTCTGTCAGCGGCAGCTCTCAGTTTCGCGGCACCTGTGGCTCATGGGGGAGACCACCGCCACCACCATCACCACCAACATCGTGGGACCACCGCGGCGCCTACGACCACGACCGTCACATCACCGGAAACTATAACGGCTGACGCGTCTCTGGGAGATCTGCTGGACCTGAGCCTGCCTCCGGTGAAGGTAGAAGGATCCAAATTGACTATTGGCGACGGCTGGAAGGAAGGCGAACAGAGTCTTGAAGGAACAACCGTGGTTGGTGTCGATATACAGGGCAAGCACTACGACCTGAGACTTCCGGCGTCTGTGGAAAACGCTTTCGACCCTGAGCAGCGGGAGGCAATAATCGGCGTTTTCAAGAACGGTCCGAGTACAGAGGGGAATGTGTCAGTCTGGGGCGCCGGTGGTGATTCATCGGAATATACGCGTTGGCAAATTGAAGACCATACACACGTGTTCCATATGCCTCAAGCAGTGCTCAGTGTTCTCGACCCTGACAGCGAAACAAGTGACCCTTCTGCAGACAGCGAACTGGACTTGCTCACCTTCGCCTTGGCATTCGCCGTCGAGAAAGGTACCGGGCCTACGCGCACAGACTACGATAAGGCCGCGGAACTCGACCTTCAGTGGGTTCTGAAGACGGAAATCAAGAACCATCTCAAACAAATTCAAACCTTAGTGGAACTCCTATAG
